# Ambient temperature and mortality due to acute myocardial infarction in Brazil: an ecological study of time-series analyses

**DOI:** 10.1038/s41598-019-50235-8

**Published:** 2019-09-24

**Authors:** Letícia de Castro Martins Ferreira, Mário Círio Nogueira, Ricardo Vela de Britto Pereira, William Cossich Marcial de Farias, Moreno Magalhaes de Souza Rodrigues, Maria Teresa Bustamante Teixeira, Marilia Sá Carvalho

**Affiliations:** 1 Medical Internship Department, School of Medicine, Federal University of Juiz de Fora, Juiz de Fora, MG Brazil; 2 Public Health Department, School of Medicine, Federal University of Juiz de Fora, Juiz de Fora, MG Brazil; 3 Aeronautics Administrative Support Center, Brazilian Air Force, Rio de Janeiro, RJ Brazil; 4 Department of Physics and Astronomy, University of Bologna, Bologna, Italy; 5 Laboratório de Análise e Visualização de Dados, Fiocruz-RO, Porto Velho, Rondônia Brazil; 6 Oswaldo Cruz Foundation, Scientific Computing Program, Rio de Janeiro, RJ Brazil

**Keywords:** Risk factors, Cardiology

## Abstract

Ambient temperature may lead to decompensation of cardiovascular diseases and deaths by acute myocardial infarction (AMI). Little is known about this relationship in South American countries located in regions of a hot climate. This study aims to investigate the effects of ambient temperature on mortality due to AMI in six Brazilian micro-regions, which present different climates. We analyzed daily records of deaths by AMI between 1996 and 2013. We estimated the accumulate relative and attributable risks with lags of up to 14 days, using distributed non-linear lag model. Micro-regions that were closest to the equator did not show an association between temperature and mortality. The lowest risk temperatures varied between 22 °C and 28 °C, in the Southern region of Brazil and the Midwest region, respectively. Low temperatures associated with the highest mortality risk were observed in the same areas, varying between 5 °C and 15 °C. The number of deaths attributed to cold temperatures varied from 176/year in Brasilia to 661/year in São Paulo and those deaths attributed to hot temperatures in Rio de Janeiro amounted to 115/year. We showed the relative risk and the attributable risk of warmer and colder days in tropical regions. The estimate of the number of deaths due to climate, varying according to each area, is a way of bringing information to those responsible for health policies based on easily-understood measurements.

## Introduction

Global climate changes and climate variability over the year in the different regions of the planet affect the health state of human populations^1^.

The relationship between climate and health has been unevenly studied. The scientific literature on the effects of climate change on cardiovascular diseases (CVD), the leading cause of death worldwide, is less investigated than that on infectious diseases, especially dengue and malaria in low and middle-income countries^2,3^. Despite the public health importance of CVD in these countries, few studies evaluate climate variability and its impact on these diseases^4^. Furthermore, these effects are influenced by the capacity of adaptation, by socio-economic conditions and by health service access, and they may broaden health inequalities and vulnerabilities^3,5–7^.

A better understanding of the relationship between temperature and mortality is crucial to establish local intervention strategies to deal with temperature effects. Public health policies, derived from those studies, will be able to strengthen resilience and climate adaptability, necessary to deal with climate change consequences. Among possible interventions, the development of warning and surveillance systems, alerting the susceptible population and health services about the temperature ranges associated with higher mortality, are effective in many countries^8^.

Brazil, with its continental dimensions, encompasses the different climates: equatorial/tropical in the North and North-east; tropical in the Southeast and Center-West; and subtropical in the South. This climate variability in the different regions characterizes different exposures to heat and cold and the risk of illness.

This study aims to investigate the relationship between ambient temperature and mortality due to acute myocardial infarction (AMI) in six metropolitan areas, localized in different Brazilian regions. Measures of association and population impact were estimated to contribute to establishing guidelines for the development or early warning systems, adequate to each temperature range observed.

## Methods

### Study design and population

It is a time-series study, in which we estimated the impact of variation of average ambient air temperature on daily mortality due to AMI. We selected six metropolitan areas, defined as integrated micro-regions by the Census Bureau, localized in the five macro-regions of Brazil: Manaus (North), Recife (North-east), Rio de Janeiro (Southeast), São Paulo (Southeast), Federal District (Center-West) and Porto Alegre (South) (Fig. S1), encompassing 64 municipalities. The period of analysis was from January 1996 to December 2013, and the population at the midpoint (2004) was 35,478,625 people (Table S1). They represent the different Brazilian climate zones and have a significant number of events.

### Data

We obtained daily data on mortality due to AMI (code I21, ICD 10) and population estimates from DATASUS – Unified Health System’s Department of Informatics, accessible online^9^. Since these datasets are publicly available secondary data, the study was exempt from approval by an ethics review committee according to the resolution of the National Commission of Ethics in Research (CONEP) No.510 of 7/4/2016.

The temperature data comes from ERA-Interim Re-analysis, developed by the ECMWF (European Centre for Medium-Range Weather Forecast), available from 1979 until the present date^10^. Though data provided by weather stations are considered the gold standard for analysis, they are often not available for all regions included in analyses. The ERA-Interim re-analysis summarizes weather information from ships, airplanes, radiosondes, and satellites, which are irregularly distributed over space and time, and then integrates this information into a predictive model^11^. The use of the model’s equations enables us to extrapolate information from locally-observed parameters to unobserved parameters in a physically significant manner^10^ and have a correlation equal to or above 96% with data from weather stations, where and when these are available^12^. Estimates were applied over a uniform horizontal grid resolution, spaced 13 Km apart. For each micro-region, we calculated the daily average of the grid points located within it. Daily temperature averages are the most commonly used data in studies on climate and health^13^. Although temperature heterogeneity is present within each microregion, we supposed that, on average, the temperature varies proportionally within the region. We carried out sensitivity analyses using the minimum and maximum temperatures, instead of average temperatures (Table S2).

### Statistical analysis

In the description, we used daily and annual average rates of mortality due to AMI. We used graphs of the daily distribution with spline smoothing and boxplot graphs of the weekly counts of deaths and average annual temperature to visualize trends, seasonality, and variability of the number of deaths and the temperature data in each region.

To study the association between deaths from AMI and average temperature, we fitted generalized additive models with the negative binomial distribution. The time itself was modelled through a natural cubic spline with eight degrees of freedom per year, to adjust for the long-term trend and seasonality. The weekday was included in the models to adjust for the days in which mortality due to AMI is larger, such as weekends.

To estimate non-linear and time-lag effects, we used distributed lag non-linear models (DLNM)^14^. We selected a natural-spline with five degrees of freedom for the exposure-response function and a polynomial function with an intercept and four degrees of freedom for the lag-response function, to give the models greater flexibility. The model included estimates of up to lag 14.

For each region, we estimated the accumulated relative risks (RR) of death by AMI in set percentiles (P) of the temperature distribution concerning the optimal temperature, between lags zero and 14, corresponding to the total accumulated risk. Percentiles 2.5 and 10 were chosen to represent extreme cold and moderate cold respectively, and percentiles 90 and 97.5 to evaluate moderate heat and extreme heat.

We estimated the attributed fractions and numbers of events for non-optimal temperatures, accumulated up to lag 14, with the forward method, from the current exposure to future risks^15^. We calculated the following components:

(a) attributable risk (AR) of extreme cold (between the lowest temperature and the 2.5th percentile);

(b) AR of moderate cold (between 2.5th and 10th percentiles);

(c) AR of mild cold (between 10th percentile and minimum mortality temperature – MMT);

(d) AR of mild heat (between MMT and 90th percentile);

(e) AR of moderate heat (between 90th and 97.5th percentiles);

(f) AR of extreme heat (between 97.5th percentile and the highest temperature).

We analyzed the model residuals to detect serial autocorrelation, and we carried out sensitivity analyses for different models’ specifications, to evaluate the models’ robustness (Table S2).

The reference temperature was the point of minimum mortality risk (minimum mortality temperature – MMT for each region in the accumulated lag^16^.

We used the software R version 3.4.0, mainly using the DLNM package. R scripts are provided as supplemental material 1 (S1)^17^.

### Ethical approval

Since these datasets are publicly available secondary data, the study was exempt from approval by an ethics review committee.

## Results

We analysed 330,096 total deaths by AMI in the six regions included in the study (Fig. S1), varying between 4,547 deaths in Manaus and 131,394 deaths in São Paulo, and between 0 and 6 cases and 4 to 49 daily cases respectively (Table 1).

**Table 1:** Distribution of daily data on mortality due to acute myocardial infarction (AMI) in the Brazilian regions included in the analysis, 1996–2013.

Variables	Regions					
	Manaus	Recife	Brasília	Rio de Janeiro	São Paulo	Porto Alegre
Population^a^	1769561	3125395	2292056	11050795	13194129	3548338
**Deaths from AMI**						
Total number	4547	33523	7937	118898	131394	33797
Average annual number	253	1862	441	6605	7300	1878
Average annual rate^b^	14.28	59.59	19.24	59.77	55.33	52.92
**Daily distribution**						
Minimum value	0	0	0	2	4	0
25^th^ Percentile	0	3	0	15	16	3
Median	0	5	1	18	19	5
Average	1	5	1	18	20	5
75^th^ Percentile	1	7	2	21	23	7
Maximum value	6	18	7	45	49	18
Average daily rate	0.04	0.16	0.05	0.16	0.15	0.15

^a^Population average in the period.

^b^Rates by 100000 inhabitants.

The exploratory analysis indicates that all regions had lower temperatures in the middle of the year, except in the Amazonia region. Temperature is minimum in June, (winter), and maximum in January (summer). The weekly boxplots of the number of deaths indicate larger seasonal variation in the Southern region (Fig. S2), presenting an inverse pattern to the temperature oscillation.

There is no visible difference in the behavior of the variability over the year. In all boxplots of mortality due to AMI, the presence of outliers usually occurs for numbers of deaths that are higher than the predicted intervals, which leads to the conclusion that there are more critical risk moments, beyond the usual prediction (Fig. S2).

**Figure 1:**
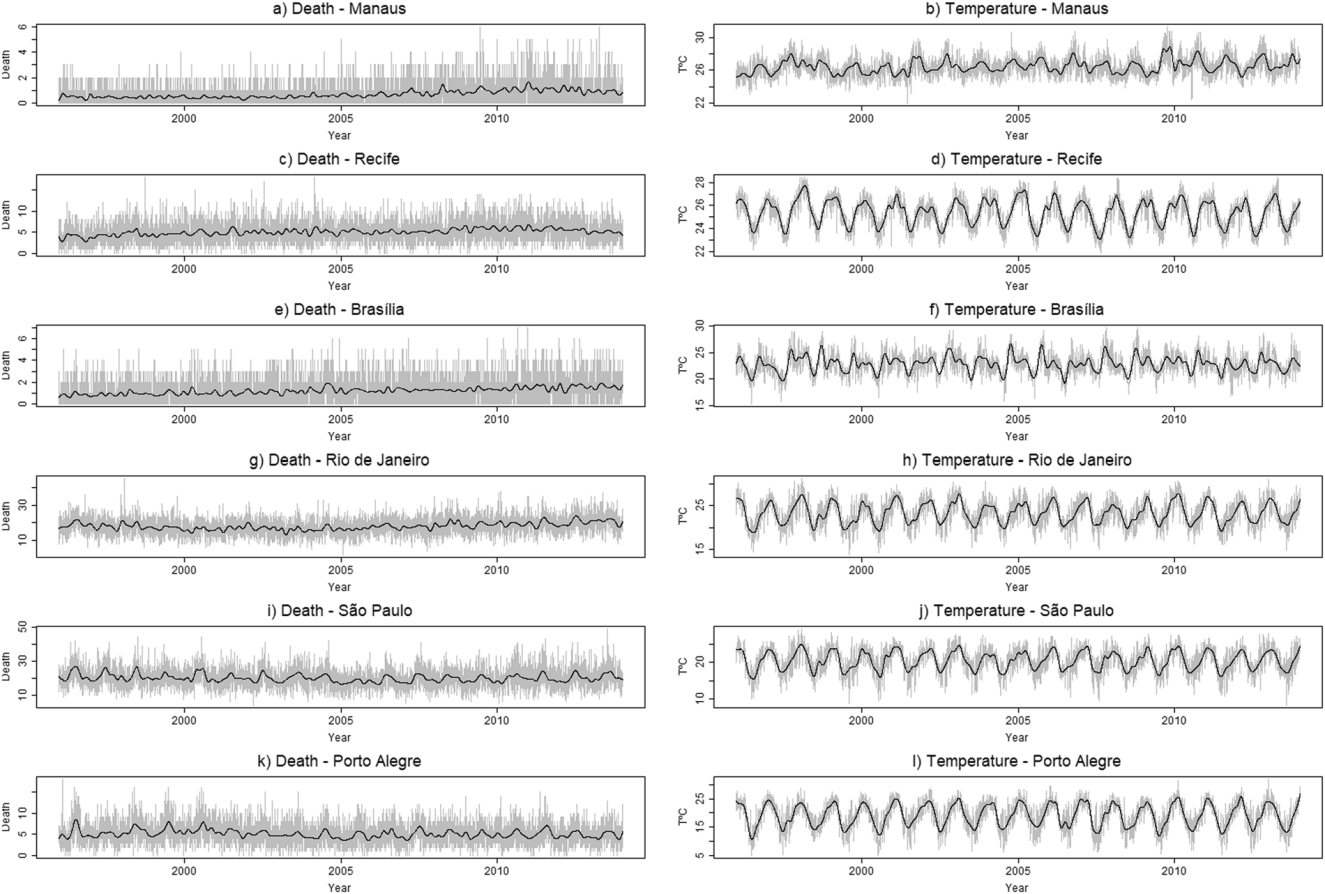
Distribution of data on mortality by acute myocardial infarction and average temperature, with superimposed time spline, for each region, 1996–2013.

There is a long-term ascending trend of death in two regions (Brasília and Manaus) not present elsewhere (Fig. 1).

Closer to the Equator, the mean temperature is higher and the overall range smaller. The minimum mortality temperatures (MMT) varied within small span, between 21.9 °C in the Southern most region and 27.8 °C in Brasília (Table 2).

**Table 2:** Distribution of daily data on average temperature (in °C) and minimum mortality temperature (MMT) in Brazilian regions included in the analysis, 1996–2013.

Distribution	Regions					
	Manaus	Recife	Brasília	Rio de Janeiro	São Paulo	Porto Alegre
Minimum value	22.0	21.8	15.3	13.8	8.4	4.9
1^st^ Percentile	24.1	22.7	18.5	16.8	12.6	7.9
2.5^th^ Percentile	24.4	23.0	19.2	17.7	13.7	9.4
10^th^ Percentile	25.1	23.7	20.4	19.5	16.1	12.5
25^th^ Percentile	25.6	24.4	21.5	21.1	18.3	15.7
Median	26.4	25.5	22.6	23.3	20.7	19.7
Average	26.5	25.4	22.8	23.3	20.5	19.1
75^th^ Percentile	27.2	26.3	24.0	25.6	22.9	22.9
90^th^ Percentile	28.0	26.9	25.5	27.2	24.4	24.8
97.5^th^ Percentile	29.1	27.4	27.0	28.4	25.8	26.4
99^th^ Percentile	29.6	27.8	27.8	29.1	26.6	27.3
Maximum value	31.3	28.5	29.7	31.3	29.0	31.9
Minimum Mortality Temperature (MMT)	25.9	24.8	27.8	26.1	23.4	21.9

There was no association between temperature and mortality due to AMI in the northern regions. In the other areas, the relative risk (RR) due to extreme cold ranged from 1.33 in Rio de Janeiro to 1.91 in Brasilia, and due to moderate cold from 1.20 in Rio de Janeiro to 1.90 in Brasilia. Only Rio de Janeiro had an association with heat, reaching a RR of 1.05 for moderate heat and 1.24 for extreme heat (Table 3). The attributable fraction of AMI deaths to cold temperatures (adding extreme, moderate and mild cold) was from 6.2% in Rio de Janeiro to 40.0% in Brasília, which corresponds to the number of deaths, respectively, 479/year and 176/year. In Rio de Janeiro, the heat was responsible for 115 deaths/year in the period (Table 4).

**Table 3:** Relative risks (with 95% confidence intervals) of death by acute myocardial infarction due to exposure to an average temperature in some regions, with each region’s minimum mortality temperature (MMT) as a reference.

Average temperature	Regions					
	Manaus	Recife	Brasília	Rio de Janeiro	São Paulo	Porto Alegre
Extreme cold	1.04 (0.65, 1.67)	1.20 (0.97, 1.48)	1.91 (1.27–2.88)	1.33 (1.24, 1.44)	1.50 (1.39, 1.63)	1.78 (1.55, 2.06)
Moderate cold	1.04 (0.71, 1.52)	1.11 (0.95, 1.29)	1.90 (1.31–2.76)	1.20 (1.13, 1.29)	1.28 (1.19, 1.37)	1.48 (1.31, 1.67)
Moderate heat	1.08 (0.80, 1.47)	1.09 (0.96, 1.24)	—	1.05 (1.03, 1.08)	1.01 (0.99, 1.03)	1.05 (0.94, 1.17)
Extreme heat	1.07 (0.73, 1.56)	1.14 (0.97, 1.33)	—	1.24 (1.16, 1.32)	1.07 (1.00, 1.15)	1.05 (0.92, 1.20)

Extreme cold: 2.5^th^ percentile of average temperature.

Moderate cold: 10^th^ percentile of average temperature.

Moderate heat: 90^th^ percentile of average temperature.

Extreme heat: 97.5^th^ percentile of average temperature.

**Table 4:** Fractions and numbers of deaths per year (with 95% confidence intervals) by acute myocardial infarction attributable to exposure to an average temperature in some regions, 1996–2013.

Variable	Average Temperature	Regions			
		Brasília	Rio de Janeiro	São Paulo	Porto Alegre
Attributable fraction (%)	Extreme cold	1.3 (0.3; 1.7)	0.7 (0.5; 0.9)	1.1 (0.9; 1.3)	1.6 (1.3; 1.8)
	Moderate cold	3.7 (1.8; 5.0)	1.7 (1.2; 2.1)	2.2 (1.8; 2.6)	3.4 (2.6; 4.1)
	Mild cold	35.0 (14.3; 49.7)	4.8 (2.0; 7.4)	5.7 (3.0; 8.6)	11.1 (6.8; 14.6)
	Mild heat	—	0.2 (0.1; 0.2)	—	—
	Moderate heat	—	0.8 (0.6; 1.0)	—	—
	Extreme heat	—	0.8 (0.6; 1.0)	—	—
	Total	40.0 (16.4; 56.4)	9.0 (5.0; 12.6)	9.0 (5.7; 12.5)	16.1 (10.7; 20.5)
Preventable deaths per year	Extreme cold	6 (2; 8)	49 (36; 60)	83 (67; 96)	30 (24; 35)
	Moderate cold	16 (8; 22)	111 (82; 138)	162 (130; 191)	64 (51; 77)
	Mild cold	154 (64; 217)	319 (118; 513)	416 (199; 630)	208 (129; 280)
	Mild heat	—	10 (3; 16)	—	—
	Moderate heat	—	52 (37; 67)	—	—
	Extreme heat	—	53 (39; 64)	—	—
	Total	176 (74; 247)	594 (315; 858)	661 (396; 917)	302 (204; 392)

Extreme cold: minimum to 2.5^th^ percentiles of average temperature.

Moderate cold: 2.5^th^ to 10^th^ percentile of average temperature.

Mild cold: 10^th^ percentile to minimum mortality temperature (MMT).

Mild heat: MMT to 90^th^ percentile of average temperature.

Moderate heat: 90^th^ to 97.5^th^ percentile of average temperature.

Extreme heat: 97.5^th^ percentile to maximum of average temperature.

**Figure 2:**
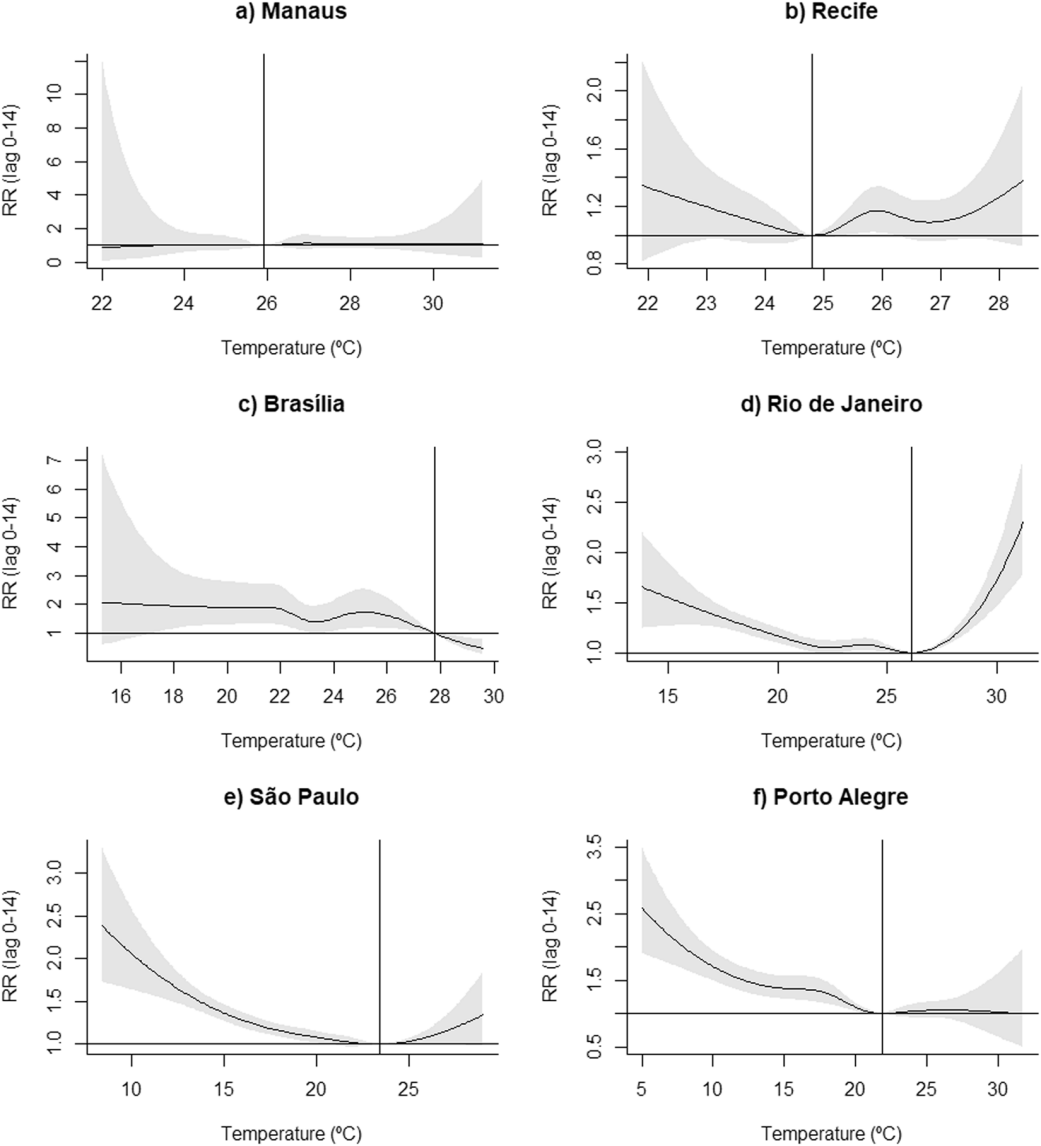
Accumulated relative risks by temperature for each region, with indications of minimal mortality temperature -MMT (solid vertical line).

**Figure 3:**
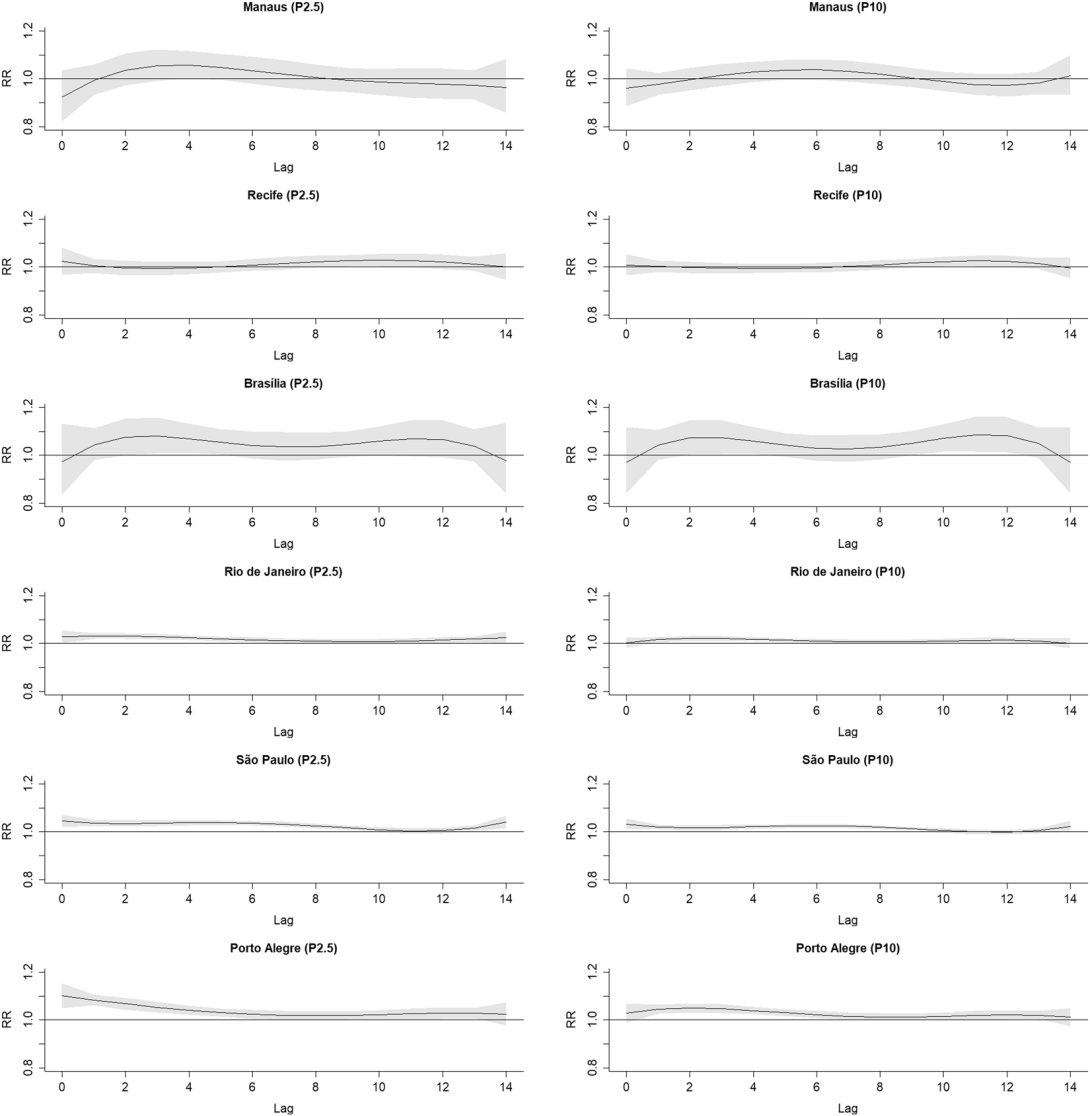
Relative risks of up to lag 14 for each region, by 2.5^th^ and 10^th^ percentiles of temperature (extreme and moderate cold).

**Figure 4:**
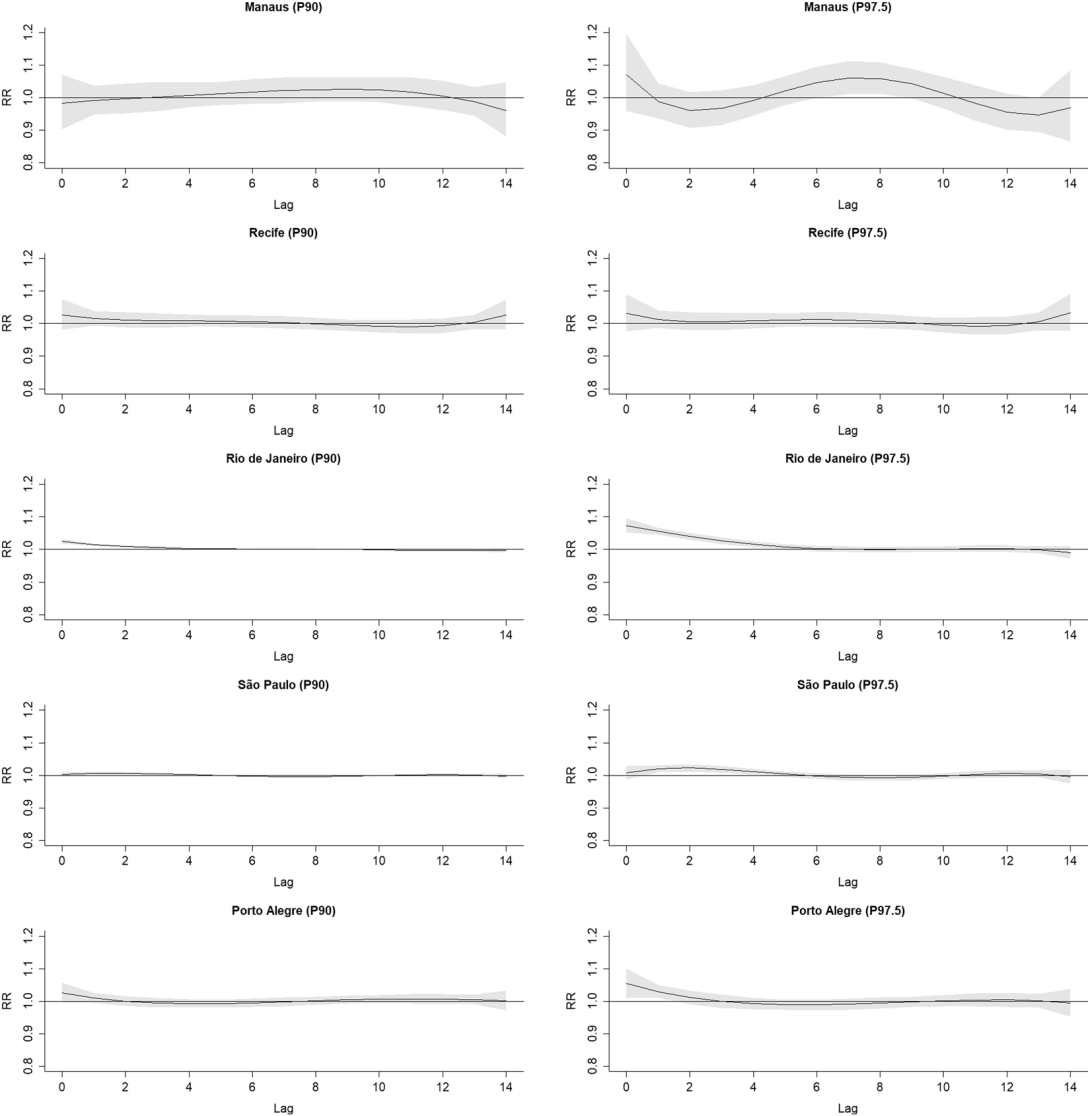
Relative risks of up to lag 14 for each region, by 90^th^ and 97.5^th^ percentiles of temperature (moderate and extreme heat).

Figure 2 shows the accumulated RR curves by temperature, with 95% confidence intervals, for each region, with vertical lines representing MMT. Figure 3 shows the RR curves per lag in extreme and moderate cold. Figure 4 does the same for extreme and moderate heat. In Manaus and Recife, no association was present in any lag. In the other regions, association with both extreme and moderate cold were present in almost all lags, and in Rio de Janeiro with moderate and extreme heat. São Paulo and Porto Alegre presented small RR with extreme heat just in the first days, with a reduction, thereafter, suggesting a displacement effect, an anticipation of deaths, called harvesting effect.

The sensitivity analysis, varying the models’ specifications, presented small differences of the RR estimates, showing the robustness of our results (Table S2).

## Discussion

In this study, we analysed six metropolitan areas in the largest country in Latin America, Brazil, including a total population of 35 million people, and all regions and climates in the country. We estimated the impact of temperature AMI mortality, adding valuable knowledge to the few studies that exist on this topic in equatorial, tropical and subtropical climates. The lack of studies in this field is probably a consequence of the perception that in tropical countries the impact of the climate on non-communicable diseases is negligible^2^. Two of the measurements used in this study– attributable risk and preventable deaths – are not commonly used^15^. They were chosen because they enable the necessary dialogue between epidemiological studies and guidelines for managers and public health practitioners.

Within Brazil, with its continental dimensions and climate diversity, the association between temperature and AMI are more substantial in regions with higher thermal amplitude. Our results are consistent with prior studies, which found peaks of mortality due to AMI during winter^18–22^.

In the regions closer to the Equator (Fig. S1), with small thermal amplitudes, there was no association between temperature and mortality due to AMI, either for cold or hot temperatures (Table 2). A study recently published investigated the relationship between temperature and hospitalizations, finding a positive association with all-cause admissions in all Brazilian regions, but minimal association with cardiovascular causes^23^. Two studies conducted in Vietnam, an equatorial and tropical climate country, presented conflicting results. The oldest one, published in 2016, showed no association between temperature and hospitalizations due to cardiovascular disease, whereas the most recent one showed association between extreme temperatures and AMI in other regions of the country^24,25^. As the regions are further away from the Equator, the associations are more consistent, both for hot and cold weather. Studies in European and Northeast Asia countries and South Korea in higher latitudes have shown greater vulnerability to extreme temperatures^26–29^. However, some systematic reviews have found diverging results, showing a reduction in this association in cities further away from the Equator^13,24^.

The AMI mortality associated with lower temperatures is related to the physiological characteristics of the cardiovascular system, particularly in older people^30–32^. This phenomenon is present even in regions where high and mild temperatures predominate almost year-round, in which the population is not acclimated. The weather acclimation is the result of a series of physiological, behavioral and technological developments, as well as external factors^33^. Colder temperatures affected the AMI mortality in the Center-West, Southeast and South regions, similarly to other areas of the world, where the mortality increase in colder temperatures extends over several weeks^27,34^.

The effect of heat on mortality was disparate in different regions^35^. In São Paulo and Porto Alegre the highest risk was observed in the first days and reversed in the following period, suggesting the harvesting effect. On the other hand, in Rio de Janeiro the risk increased until lag 14. This pattern is possibly due to the heat extending over several days, accumulating the effect, while the other regions alternate between very hot and milder days. These findings are broadly consistent with other studies in Brazil, in spite of applied to different health outcomes^36^.

The physiologic causal pathways leading to cardiovascular diseases mortality associated with heat might be related to changes in heart rate, blood viscosity and coagulability, reductions in brain perfusion and hydroelectric imbalances^31,35^. Concerning acclimation, the Brazilian population living in smaller latitudes present greater adaptability and resilience to high temperatures and, therefore, lower or absent risks. In temperate countries, a reduction in mortality due to heatwaves in recent years is attributed to public campaigns that led to the better adaptation of residences and the behaviour of the population to local climate condition^35,37^.

Another important aspect is the interaction between temperature and pollution. Studies have shown the effects of particulate material (PM) on mortality due to cardiovascular diseases are modified by the temperature^38^. In Brazil, the period of the highest temperatures is also the period of rainfall, which dilutes PM, while in winter the phenomenon of thermal inversion is common, especially in São Paulo, increasing the concentration of pollutants. Despite the undeniable importance of air pollution to the increase in mortality due to AMI^39,40^, in most Brazilian cities, access to high-quality pollution data is limited, limiting the inclusion of this variable. Additionally, our primary objective is not to isolate the effect of temperature and pollution each, but to assess the impact of their combination, as captured by temperature, information which is far more accessible^41,42^.

A strong feature of our study is the method applied – DLNM – that enabled the capture of the complex, non-linear and lagged dependencies present over the relationship between daily temperature and health event. In addition, the model allowed the estimation of both RR and attributable risk, considering the number of days and duration of those effects^4,15,20^. These models enable us to flexibly capture complex, non-linear and lagged dependencies of the exposure-response relationships through two functions that model the exposure-response and lag-response relationships, respectively^14^.

A multicenter study has applied this method to all-cause mortality in Brazilian state capitals, some of which were included in our study^43^. They found a relationship between high and low temperatures with an increase in overall mortality in some cities, including Manaus, and we did not. This divergence is probably due to the outcome analysed: all-cause versus AMI deaths. In the North region, mortality due to infectious diseases, especially childhood diarrhoea, is critical and its relationship to temperature is well-established^44^. Besides, the number of daily deaths in Manaus is small and, consequently, the statistical power is lower. In addition, another recent study showed an association between heat exposure and increased risk of all-causes hospitalizations in all Brazilian regions, but not for cardiovascular hospitalizations^45,46^.

Despite the relevance of studying climate risk factors for overall mortality, to refine this analysis for specific causes is relevant to propose specific prevention actions. We believe that our results will contribute to settling the scientific basis for stakeholders towards a policy for mitigating climate effects on health. In France and Hong Kong, after heatwaves that culminated in several deaths, a set of measures were implemented, using the weather forecasts to anticipate the event. Brazilian metropolises could adapt some of those interventions to prevent deaths caused by weather events^47,48^.

To develop a warning protocol, the health system must take into account the temperature effects in each region. In Rio de Janeiro, temperatures under 19.5 °C, considered to be moderately cold, and above 27.2 °C, which corresponds to moderate heat, already lead to an increased risk of death. In Porto Alegre, São Paulo and Brasília, only moderate cold was associated with the risk of death, at the temperatures of 12,5 °C, 16.1 °C and 20.4 °C, respectively.

Brazil is an unequal country, both in terms of socio-economic conditions and of health services access. Temperature is a widely-accessible climate element, available even in the most remote areas of the country. In the context of global climate change, the number of deaths attributed to a specific temperature ranges is a key element to establish local guidelines. In the primary health care services, adequate orientation, especially of the elderly, is a simple and effective measure. Besides, preparedness for climate-driven health events in secondary and tertiary health units, including emergency rooms, can be strengthened. This study contributes to the discussion of public policies and actions in different social sectors directed at the reduction of damage caused by adverse climate.

## Supplementary information

Supplementary Information - AMBIENT TEMPERATURE AND MORTALITY DUE TO ACUTE MYOCARDIAL INFARCTION IN BRAZIL: AN ECOLOGICAL STUDY WITH TIME-SERIES ANALYSES

## Data Availability

The data on mortality due to AMI (code I21, CID 10) and population estimates data used in preparation of this article were obtained from DATASUS – Unified Health System’s Department of Informatics, accessible online (http://datasus.saude.gov.br/) and the temperature data comes from ERA-Interim Re-analysis, developed by the ECMWF (European Centre for Medium-Range Weather Forecast)Era Interim accessible online (https://www.ecmwf.int/en/forecasts/datasets/reanalysis-datasets/era-interim).
