# Supplementary material for: Ambient temperature and mortality due to acute myocardial infarction in Brazil: an ecological study of time-series analyses
Source: Sci Rep. 2019 Sep 24;9:13790. doi: 10.1038/s41598-019-50235-8 (PMC6760184; doi:10.1038/s41598-019-50235-8)
Supplement: Supplementary file 1 — Supplementary Information - AMBIENT TEMPERATURE AND MORTALITY DUE TO ACUTE MYOCARDIAL INFARCTION IN BRAZIL: AN ECOLOGICAL STUDY WITH TIME-SERIES ANALYSES [file 41598_2019_50235_MOESM1_ESM.pdf]

Supplementary Information

AMBIENT TEMPERATURE AND MORTALITY DUE TO ACUTE MYOCARDIAL INFARCTION IN BRAZIL: AN ECOLOGICAL STUDY WITH TIME-SERIES ANALYSES

Letícia de Castro Martins Ferreira, Mário Círio Nogueira, Ricardo Vela de Britto Pereira, William Cossich Marcial de Farias, Moreno Magalhaes de Souza Rodrigues, Maria Teresa Bustamante Teixeira, Marilia Sá Carvalho.

Table of Contents

S1: R Script.

Figure S1 – Geographic locations of micro-regions included in the analysis.

Figure S2 – Weekly boxplots of the distribution of deaths by acute myocardial infarction and average temperatures in the regions over the course of a year.

Table S1: Municipalities that integrate the microregions and their populations in the year 2004.

Table S2 - Sensitivity analysis, varying specifications of the models, with estimation of the relative risk accumulated until the lag 14.

S1: R Script.

```
##################################################################################
##  SCRIPT: "AMBIENT TEMPERATURE AND MORTALITY DUE TO ACUTE                    ##
## MYOCARDIAL INFARCTION IN BRAZIL: AN ECOLOGICAL STUDY                        ##
## WITH TIME-SERIES ANALYSES"                                                   ##
##################################################################################

### DESCRIPTIVE STATISTICS

# daily distribution of deaths
round(summary(banco$COUNT))
boxplot(banco$COUNT)
hist(banco$COUNT)

# total sum of death
sum(banco$COUNT)

# annual average of death
round(sum(banco$COUNT)/18)

# Average annual rates per 100000 inhabitants
round((sum(banco$COUNT)/18)/mean(banco$pop, na.rm =T)*100000,2)

# average daily temperature distribution
round(summary(banco$temp_med),1)
boxplot(banco$temp_med)
hist(banco$temp_med)

# boxplot of variables per week
library(lubridate)
banco$semana <- week(banco$Data)
boxplot(COUNT~semana,data=banco,main="",xlab="Week",ylab="Death")
boxplot(temp_med~semana,data=banco,main="",xlab="Week",ylab="TºC")
```

```
# temporal graphs of deaths and temperatures
plot(banco$tempo,banco$COUNT,main = "Death",type="l")
plot(banco$tempo,banco$temp_med, main = "Temperature",type="l")

### EXPLORATORY STATISTICS

# generalized additive model with natural spline of time
library(mgcv)
library(splines)
fit.death <- gam(COUNT~ns(tempo,8*18),family=nb,data=banco)
plot(predict(fit.death,type="response"),type="l",main = "Death")
fit.temp <- gam(temp_med~ns(tempo,8*18),family=nb,data=banco)
plot(predict(fit.temp,type="response"), type="l",main = "Temperature")

# adding the temperature
library(mgcv)
mod <- gam(COUNT ~ temp_med + ns(tempo,18*8),family=nb(), data=banco)
summary(mod)
res <- residuals(mod,type="response")
plot(banco$tempo,res)
abline(h=1,lty=2,lwd=2)
pacf(res,lag.max = 14,na.action=na.pass)

# adding the day of the week
mod <- gam(COUNT ~ temp_med + ns(tempo,18*8) + temp_med + weekday,
           family=nb(), data=banco)
summary(mod)
res <- residuals(mod,type="response")
plot(banco$tempo,res)
abline(h=1,lty=2,lwd=2)
pacf(res,lag.max = 14,na.action=na.pass)
```

```
### DLNM MODELS

# main exposure
temp = banco$temp_med

# average temperature values
round(mean(temp),1)

# temperature quantis
round(quantile(temp, probs = c(0,0.01,0.025,0.1,0.25,0.5,0.75,0.9,0.975,0.99,1)),1)
P0 = round(quantile(temp,probs=0),1)
P1 = round(quantile(temp,probs=0.01),1)
P2.5 = round(quantile(temp,probs=0.025),1)
P10 = round(quantile(temp,probs=0.1),1)
P25 = round(quantile(temp,probs=0.25),1)
P50 = round(quantile(temp,probs=0.5),1)
P75 = round(quantile(temp,probs=0.75),1)
P90 = round(quantile(temp,probs=0.9),1)
P97.5 = round(quantile(temp,probs=0.975),1)
P99 = round(quantile(temp,probs=0.99),1)
P100 = round(quantile(temp,probs=1),1)

## Creating cross-basis objects for Temperature (lags up to 14 days)
library(dlnm)
cb <- crossbasis(temp, lag=14, argvar=list(fun="ns",df=5),
                 arglag=list(fun="poly",degree=4))

## adjusting the GAM / DLMN model with negative binomial distribution
library(mgcv)
library(splines)
mod <- gam(COUNT~cb+ns(tempo,18*8)+weekday,family=nb,data=banco)
res.mod <- resid(mod, type = "deviance")
par(mar=c(4,4,4,4),mfrow=c(1,1))
plot(res.mod)
```

```
lines(lowess(res.mod),col="red")
library(stats)
pacf(res.mod,lag.max = 14,na.action=na.pass)

## ESTIMATING THE MINIMUM RISK TEMPERATURE
# Ref: Tobías et al., 2017.
source("findmin.R")
MMT <- findmin(cb,mod,from=P1,to=P99)
print(MMT)

## Prediction model with minimum risk temperature as reference
pred <- crosspred(basis=cb,model=mod,from=P0,to=P100,by=0.1,cumul=TRUE,cen=MMT)

## Plot cumulative RR for temperature range
par(mar=c(4,4,4,4),mfrow=c(1,1))
plot(pred,"slices",cumul=TRUE,lag=14,xlab="Temperature (ºC)",ylab="RR (lag 0-14)",main="")
abline(v=MMT)

## Plot RR at specific temperature for lag range
par(mar=c(4,4,4,4),mfrow=c(2,2))
plot(pred,"slices",var=as.character(P2.5),lty=1,cumul=F,ylab="RR",
    main=paste("Lag-response curve at ",P2.5,"ºC","(P2.5)"))
plot(pred,"slices",var=as.character(P10),lty=1,cumul=F,ylab="RR",
    main=paste("Lag-response curve at ",P10,"ºC","(P10)"))
plot(pred,"slices",var=as.character(P90),lty=1,cumul=F,ylab="RR",
    main=paste("Lag-response curve at ",P90,"ºC","(P90)"))
plot(pred,"slices",var=as.character(P97.5),lty=1,cumul=F,ylab="RR",
    main=paste("Lag-response curve at ",P97.5,"ºC","(P97.5)"))

## RR acumulated total for the entire period for percentiles 2.5/10/90/97.5
round(cbind(pred$allRRfit,pred$allRRlow,pred$allRRhigh)[as.character(P2.5),],2)
round(cbind(pred$allRRfit,pred$allRRlow,pred$allRRhigh)[as.character(P10),],2)
round(cbind(pred$allRRfit,pred$allRRlow,pred$allRRhigh)[as.character(P90),],2)
round(cbind(pred$allRRfit,pred$allRRlow,pred$allRRhigh)[as.character(P97.5),],2)
```

```
### CALCULATE FRACTIONS ATTRIBUTABLE TO EXPOSURE
# Ref: Gasparrini and Leone, 2014.
source("attrdl.R")

# fraction attributable to extreme cold (between P0 and P2.5) in percentual
round(attrdl(temp,cb,banco$COUNT,mod,type="af",cen=MMT,
        range=c(P0,P2.5),dir="forw")*100,1)
afsim.frio <- attrdl(temp,cb,banco$COUNT,mod,type="af",cen=MMT,
        range=c(P0,P2.5),sim=T,nsim=1000,dir="forw")
round(quantile(afsim.frio,c(2.5,97.5)/100)*100,1)

# fraction attributable to moderate cold (between P2.5 and P10)
round(attrdl(temp,cb,banco$COUNT,mod,type="af",cen=MMT,
        range=c(P2.5,P10),dir="forw")*100,1)
afsim.frio <- attrdl(temp,cb,banco$COUNT,mod,type="af",cen=MMT,
        range=c(P2.5,P10),sim=T,nsim=1000,dir="forw")
round(quantile(afsim.frio,c(2.5,97.5)/100)*100,1)

# fraction attributable to mild cold (between P10 and MMT)
round(attrdl(temp,cb,banco$COUNT,mod,type="af",cen=MMT,
        range=c(P10,MMT),dir="forw")*100,1)
afsim.frio <- attrdl(temp,cb,banco$COUNT,mod,type="af",cen=MMT,
        range=c(P10,MMT),sim=T,nsim=1000,dir="forw")
round(quantile(afsim.frio,c(2.5,97.5)/100)*100,1)

# fraction attributable to mild heat (between MMT and P90)
round(attrdl(temp,cb,banco$COUNT,mod,type="af",cen=MMT,
        range=c(MMT,P90),dir="forw")*100,1)
afsim.calor <- attrdl(temp,cb,banco$COUNT,mod,type="af",cen=MMT,
        range=c(MMT,P90),sim=T,nsim=1000,dir="forw")
round(quantile(afsim.calor,c(2.5,97.5)/100)*100,1)

# fraction attributable to moderate heat (between P90 and P97.5)
```

```
round(attrdl(temp,cb,banco$COUNT,mod,type="af",cen=MMT,
      range=c(P90,P97.5),dir="forw")*100,1)
afsim.calor <- attrdl(temp,cb,banco$COUNT,mod,type="af",cen=MMT,
      range=c(P90,P97.5),sim=T,nsim=1000,dir="forw")
round(quantile(afsim.calor,c(2.5,97.5)/100)*100,1)

# fraction attributable to extreme heat (between P97.5 and P100)
round(attrdl(temp,cb,banco$COUNT,mod,type="af",cen=MMT,
      range=c(P97.5,P100),dir="forw")*100,1)
afsim.calor <- attrdl(temp,cb,banco$COUNT,mod,type="af",cen=MMT,
      range=c(P97.5,P100),sim=T,nsim=1000,dir="forw")
round(quantile(afsim.calor,c(2.5,97.5)/100)*100,1)


### CALCULATE NUMBERS ATTRIBUTABLE TO EXPOSURE

# number attributable to extreme cold (between P0 and P2.5)
round(attrdl(temp,cb,banco$COUNT,mod,type="an",cen=MMT,
      range=c(P0,P2.5),dir="forw"))
afsim.frio <- attrdl(temp,cb,banco$COUNT,mod,type="an",cen=MMT,
      range=c(P0,P2.5),sim=T,nsim=1000,dir="forw")
round(quantile(afsim.frio,c(2.5,97.5)/100))

# number attributable to moderate cold (between P2.5 and P10)
round(attrdl(temp,cb,banco$COUNT,mod,type="an",cen=MMT,
      range=c(P2.5,P10),dir="forw"))
afsim.frio <- attrdl(temp,cb,banco$COUNT,mod,type="an",cen=MMT,
      range=c(P2.5,P10),sim=T,nsim=1000,dir="forw")
round(quantile(afsim.frio,c(2.5,97.5)/100))

# number attributable to mild cold (between P10 and MMT)
round(attrdl(temp,cb,banco$COUNT,mod,type="an",cen=MMT,
      range=c(P10,MMT),dir="forw"))
afsim.frio <- attrdl(temp,cb,banco$COUNT,mod,type="an",cen=MMT,
```

```
    range=c(P10,MMT),sim=T,nsim=1000,dir="forw")
round(quantile(afsim.frio,c(2.5,97.5)/100))

# number attributable to mild heat (between MMT and P990)
round(attrdl(temp,cb,banco$COUNT,mod,type="an",cen=MMT,
    range=c(MMT,P90),dir="forw"))
afsim.calor <- attrdl(temp,cb,banco$COUNT,mod,type="an",cen=MMT,
    range=c(MMT,P90),sim=T,nsim=1000,dir="forw")
round(quantile(afsim.calor,c(2.5,97.5)/100))

# number attributable to moderate heat (between P90 and P97.5)
round(attrdl(temp,cb,banco$COUNT,mod,type="an",cen=MMT,
    range=c(P90,P97.5),dir="forw"))
afsim.calor <- attrdl(temp,cb,banco$COUNT,mod,type="an",cen=MMT,
    range=c(P90,P97.5),sim=T,nsim=1000,dir="forw")
round(quantile(afsim.calor,c(2.5,97.5)/100))

# number attributable to extreme heat (between P97.5 and P100)
round(attrdl(temp,cb,banco$COUNT,mod,type="an",cen=MMT,
    range=c(P97.5,P100),dir="forw"))
afsim.calor <- attrdl(temp,cb,banco$COUNT,mod,type="an",cen=MMT,
    range=c(P97.5,P100),sim=T,nsim=1000,dir="forw")
round(quantile(afsim.calor,c(2.5,97.5)/100))

### SENSITIVITY ANALYSIS / MODEL COMPARISONS

## original cross-basis (lags up to 14 days, df = 25)
cb <- crossbasis(temp,lag=14,argvar=list(fun="ns",df=5),arglag=list(fun="poly",degree=4)) #1
# modifying the number of df
cb <- crossbasis(temp,lag=14,argvar=list(fun="ns",df=3),arglag=list(fun="poly",degree=4)) #2
cb <- crossbasis(temp,lag=14,argvar=list(fun="ns",df=7),arglag=list(fun="poly",degree=4)) #3
cb <- crossbasis(temp,lag=14,argvar=list(fun="ns",df=5),arglag=list(fun="poly",degree=2)) #4
cb <- crossbasis(temp,lag=14,argvar=list(fun="ns",df=5),arglag=list(fun="poly",degree=6)) #5
# modifying the number of lags
```

```
cb <- crossbasis(temp,lag=10,argvar=list(fun="ns",df=5),arglag=list(fun="poly",degree=4)) #6
cb <- crossbasis(temp,lag=18,argvar=list(fun="ns",df=5),arglag=list(fun="poly",degree=4)) #7

## original gam / dlnm model
mod <- gam(COUNT~cb+ns(tempo,18*8)+weekday,family=nb,data=banco) #1
# modifying df of time spline
mod <- gam(COUNT~cb+ns(tempo,18*4)+weekday,family=nb,data=banco) #8
mod <- gam(COUNT~cb+ns(tempo,18*12)+weekday,family=nb,data=banco) #9
# adding population to model
mod <- gam(COUNT~cb+ns(tempo,18*8)+weekday+offset(log(pop)),family=nb,data=banco) #10

## using minimum or maximum temperature instead of the mean
temp = banco$temp_min #11
temp = banco$temp_max #12

## Prediction model with minimum risk temperature as reference
P0 = round(quantile(temp,probs=0),1)
P1 = round(quantile(temp,probs=0.01),1)
P2.5 = round(quantile(temp,probs=0.025),1)
P10 = round(quantile(temp,probs=0.1),1)
P90 = round(quantile(temp,probs=0.9),1)
P97.5 = round(quantile(temp,probs=0.975),1)
P99 = round(quantile(temp,probs=0.99),1)
P100 = round(quantile(temp,probs=1),1)
source("findmin.R")
MMT <- findmin(cb,mod,from=P1,to=P99)
pred <- crosspred(cb,mod,from=P0,to=P100,by=0.1,cumul=TRUE, cen=MMT)

## RR accumulated total for the entire period for percentiles 2.5/10/90/97.5
round(cbind(pred$allRRfit,pred$allRRlow,pred$allRRhigh)[as.character(P2.5),],2)
round(cbind(pred$allRRfit,pred$allRRlow,pred$allRRhigh)[as.character(P10),],2)
round(cbind(pred$allRRfit,pred$allRRlow,pred$allRRhigh)[as.character(P90),],2)
round(cbind(pred$allRRfit,pred$allRRlow,pred$allRRhigh)[as.character(P97.5),],2)
```

Figure S1 – Geographic locations of micro-regions included in the analysis.

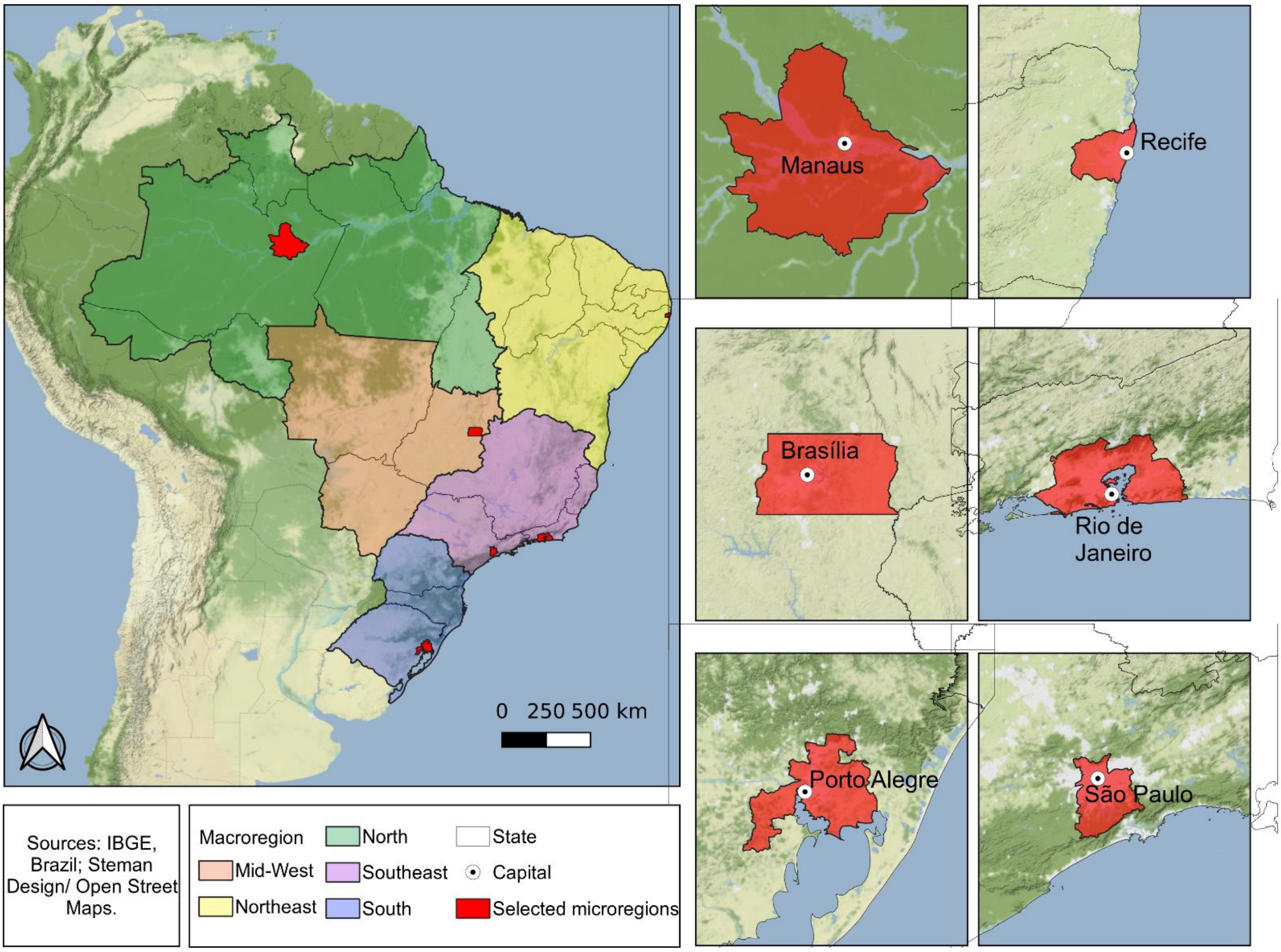

Note: Map tiles by Stamen Design (http://stamen.com/), under CC BY 3.0 (http://creativecommons.org/licenses/by/3.0). Data by OpenStreetMap (http://openstreetmap.org/), under ODbL (http://www.openstreetmap.org/copyright).

Figure S2 – Weekly boxplots of the distribution of deaths by acute myocardial infarction and average temperatures in the regions over the course of a year.

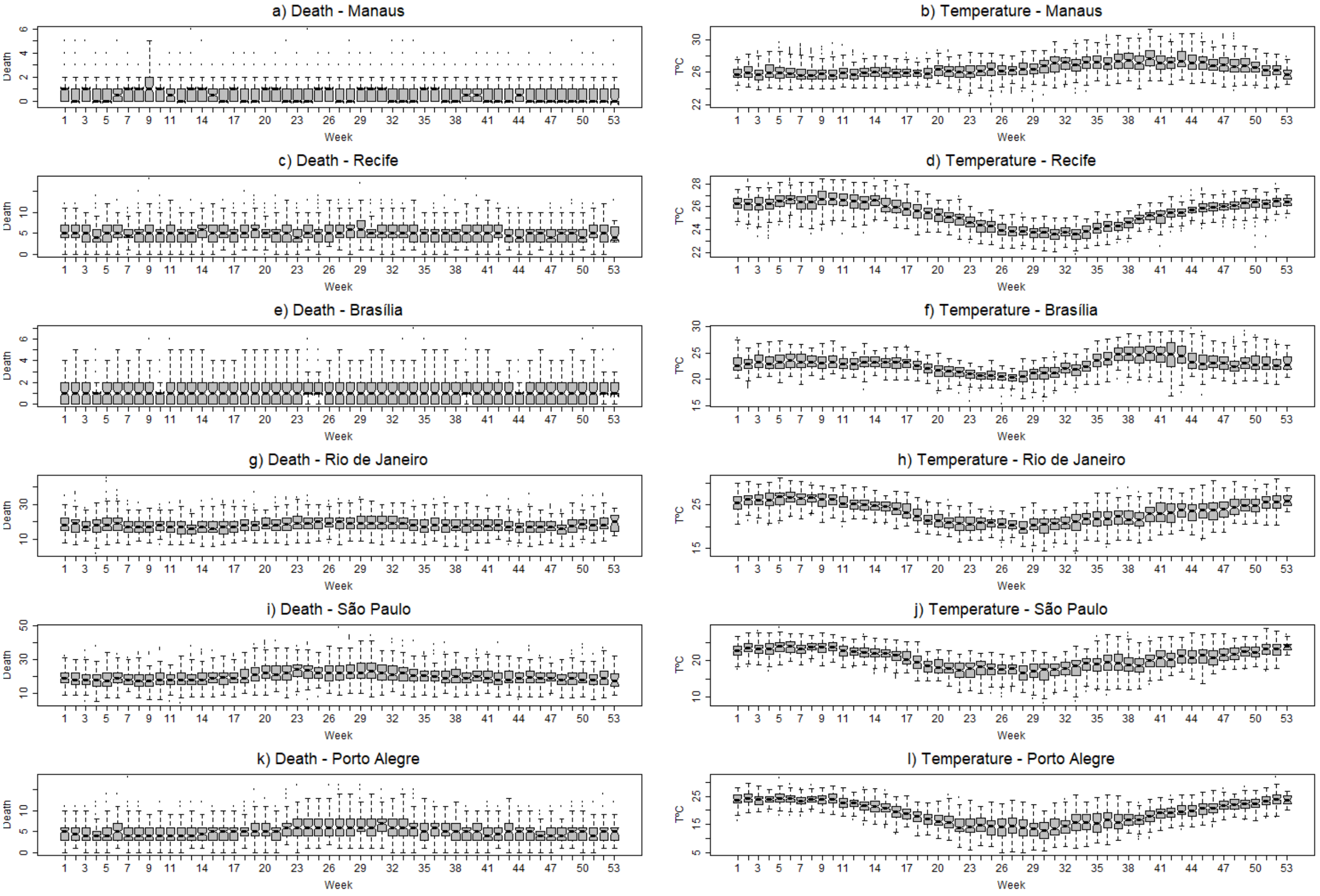

Table S1: Municipalities that integrate the microregions and their populations in the year 2004.

Regions	Micro-regions	Climate Types[a]	Municipalities	Latitude (º N)	Longitude (º E)	Altitude (meters)	Population (2004)
North	Manaus	Equatorial	Manaus	-03º.06' 07"	-60º. 01' 30"	92	1.565.709
			Careiro				25.825
			Careiro da Várzea				16.905
			Iranduba				37.746
			Manacapuru				80.395
			Manaquiri				13.518
			Autazes				27.277
			Total				1.767.375
Northeast	Recife	Tropical mar-itime	Recife	-08 03' 14"	-34º. 52' 52"	4	1.473.461
			Camaragibe				140.577
			Jaboatão dos Guararapes				619.845
			Moreno				53.287
			Olinda				378.649
			Paulista				282.811
			Abreu e Lima				93.907
			São Lourenço da Mata				92.244
			Total				3.134.781
Center-West	Brasília	Tropical continental	Brasília	-15º. 46' 47"	- 47º. 55'47"		Center-West
Southeast	Rio de Janeiro	Tropical coastal	Rio de Janeiro	-22º. 54' 10"	-43º. 12' 27"	2	6.010.814
			Duque de Caxias				819.096
			Guapimirim				41.959
			Itaboraí				205.857
			Japeri				90.370
			Magé				222.930
			Maricá				88.978
			Mesquita				176.643

			Nilópolis				151.937
			Niterói				468.897
			Nova Iguaçu				804.044
			Queimados				131.388
			Belford Roxo				464.386
			São Gonçalo				936.239
			São João de Meriti				459.084
			Tanguá				28.273
			Total				11.100.895
Southeast	São Paulo	Tropical humid	São Paulo	-23º. 32’ 51”	-46º.38’ 10”	760	10.753.768
			Mauá				391.119
			Ribeirão Pires				112.382
			Rio Grande da Serra				40.006
			Santo André				662.444
			São Bernardo do Campo				758.430
			São Caetano do Sul				136.364
			Diadema				378.057
			Total				13.232.570
South	Porto Alegre	Subtropical	Porto Alegre	-29º. 59’ 24”	-51º. 05’ 01”	3	1.404.670
			Araricá				4.466
			Cachoeirinha				115.415
			Campo Bom				56.509
			Canoas				321.027
			Eldorado do Sul				31.148
			Estância Velha				37.949
			Esteio				83.900
			Glorinha				6.130
			Gravataí				253.546
			Guaíba				100.619
			Mariana Pimentel				4.003

Nova Hartz	17.207
Nova Santa Rita	18.131
Novo Hamburgo	248.569
Parobé	49.958
Alvorada	200.967
São Leopoldo	203.942
Sapiranga	74.567
Sapucaia do Sul	129.998
Sertão Santana	5.439
Viamão	246.377
Total	3.614.537

[a] Köppen’s climate classification.

Table S2 - Sensitivity analysis, varying specifications of the models, with estimation of the relative risk accumulated until the lag 14.

	Regions					
Models	Manaus	Recife	Brasília	Rio de Janeiro	São Paulo	Porto Alegre
Extreme cold						
Model 1[a]	1.04 (0.65, 1.67)	1.20 (0.97, 1.48)	1.91 (1.27, 2.88)	1.33 (1.24, 1.44)	1.50 (1.39, 1.63)	1.78 (1.55, 2.06)
Model 2[b]	1.02 (0.78, 1.32)	1.17 (0.96, 1.43)	1.58 (1.10, 2.28)	1.31 (1.22, 1.40)	1.48 (1.38, 1.59)	1.71 (1.48, 1.97)
Model 3[c]	1.05 (0.65, 1.69)	1.21 (0.97, 1.50)	1.68 (1.10, 2.56)	1.34 (1.24, 1.45)	1.51 (1.39, 1.65)	1.81 (1.53, 2.15)
Model 4[d]	1.07 (0.59, 1.96)	1.20 (0.97, 1.48)	1.95 (1.31, 2.92)	1.33 (1.23, 1.43)	1.49 (1.38, 1.62)	1.79 (1.55, 2.06)
Model 5[e]	1.05 (0.66, 1.68)	1.20 (0.97, 1.48)	1.95 (1.30, 2.94)	1.32 (1.22, 1.42)	1.51 (1.40, 1.64)	1.82 (1.58, 2.10)
Model 6[f]	1.12 (0.80, 1.61)	1.13 (0.96, 1.34)	1.73 (1.22, 2.44)	1.24 (1.17, 1.32)	1.42 (1.33, 1.52)	1.66 (1.48, 1.88)
Model 7[g]	1.04 (0.82, 1.32)	1.18 (0.94, 1.49)	1.62 (1.01, 2.60)	1.36 (1.24, 1.49)	1.56 (1.42, 1.72)	1.85 (1.56, 2.20)
Model 8[h]	1.35 (0.83, 2.20)	1.11 (1.01, 1.23)	1.67 (1.20, 2.32)	1.34 (1.25, 1.42)	1.51 (1.40, 1.62)	1.76 (1.55, 1.99)
Model 9[i]	1.06 (0.85, 1.33)	1.29 (0.98, 1.69)	2.02 (1.27, 3.23)	1.29 (1.18, 1.42)	1.45 (1.32, 1.60)	1.84 (1.55, 2.19)
Model 10[j]	1.04 (0.65, 1.67)	1.20 (0.97, 1.48)	1.91 (1.27, 2.87)	1.33 (1.24, 1.44)	1.50 (1.39, 1.63)	1.78 (1.55, 2.06)
Model 11h[k]	1.07 (0.86, 1.33)	1.04 (0.95, 1.14)	1.63 (1.04, 2.55)	1.32 (1.22, 1.43)	1.48 (1.36, 1.61)	1.73 (1.46, 2.05)
Model 12[l]	1.26 (0.74, 2.15)	1.12 (0.93, 1.35)	1.84 (1.24, 2.74)	1.27 (1.18, 1.36)	1.49 (1.37, 1.62)	1.74 (1.50, 2.01)
Moderate cold						
Model 1[a]	1.04 (0.71, 1.52)	1.11 (0.95, 1.29)	1.90 (1.31, 2.76)	1.20 (1.13, 1.29)	1.28 (1.19, 1.37)	1.48 (1.31, 1.67)
Model 2[b]	1.00 (0.98, 1.03)	1.06 (0.96, 1.17)	1.52 (1.12, 2.05)	1.20 (1.13, 1.26)	1.27 (1.19, 1.36)	1.51 (1.32, 1.73)

Model 3[c]	1.20<br>(0.76, 1.90)	1.12<br>(0.94, 1.34)	1.82<br>(1.25, 2.65)	1.21<br>(1.12, 1.30)	1.28<br>(1.19, 1.39)	1.49<br>(1.26, 1.75)
Model 4[d]	1.02<br>(0.58, 1.78)	1.11<br>(0.95, 1.30)	1.96<br>(1.35, 2.82)	1.21<br>(1.13, 1.29)	1.27<br>(1.18, 1.36)	1.50<br>(1.32, 1.69)
Model 5[e]	1.05<br>(0.72, 1.53)	1.11<br>(0.95, 1.30)	1.94<br>(1.34, 2.82)	1.19<br>(1.11, 1.27)	1.28<br>(1.19, 1.37)	1.50<br>(1.32, 1.69)
Model 6[f]	1.05<br>(0.80, 1.38)	1.05<br>(0.94, 1.19)	1.71<br>(1.24, 2.34)	1.16<br>(1.10, 1.22)	1.26<br>(1.18, 1.33)	1.42<br>(1.28, 1.58)
Model 7[g]	1.09<br>(0.64, 1.88)	1.06<br>(0.92, 1.23)	1.61<br>(1.05, 2.48)	1.25<br>(1.16, 1.36)	1.32<br>(1.21, 1.43)	1.51<br>(1.30, 1.75)
Model 8[h]	1.33<br>(0.84, 2.10)	1.07<br>(0.97, 1.17)	1.61<br>(1.18, 2.20)	1.19<br>(1.13, 1.26)	1.32<br>(1.24, 1.40)	1.44<br>(1.30, 1.59)
Model 9[i]	1.15<br>(0.69, 1.94)	1.24<br>(1.00, 1.54)	1.98<br>(1.28, 3.07)	1.16<br>(1.08, 1.25)	1.27<br>(1.17, 1.38)	1.51<br>(1.31, 1.75)
Model 10[j]	1.04<br>(0.71, 1.51)	1.11<br>(0.95, 1.30)	1.90<br>(1.31, 2.75)	1.20<br>(1.13, 1.29)	1.28<br>(1.19, 1.37)	1.48<br>(1.31, 1.67)
Model 11h[k]	1.01<br>(0.93, 1.10)	1.10<br>(0.87, 1.41)	1.70<br>(1.12, 2.58)	1.20<br>(1.12, 1.29)	1.26<br>(1.16, 1.36)	1.49<br>(1.28, 1.75)
Model 12[l]	1.24<br>(0.75, 2.04)	1.11<br>(0.94, 1.32)	1.69<br>(1.18, 2.44)	1.16<br>(1.10, 1.23)	1.31<br>(1.21, 1.41)	1.37<br>(1.21, 1.54)
Moderate heat						
Model 1[a]	1.08<br>(0.80, 1.47)	1.09<br>(0.96, 1.24)	-	1.05<br>(1.03, 1.08)	1.01<br>(0.99, 1.03)	1.05<br>(0.94, 1.17)
Model 2[b]	1.09<br>(0.82, 1.44)	1.11<br>(0.97, 1.26)	-	1.09<br>(1.06, 1.13)	1.01<br>(0.99, 1.04)	1.00<br>(0.98, 1.03)
Model 3[c]	1.26<br>(0.84, 1.91)	1.09<br>(0.94, 1.28)	-	1.01<br>(1.00, 1.03)	1.02<br>(0.95, 1.10)	1.04<br>(0.89, 1.21)
Model 4[d]	1.09<br>(0.65, 1.85)	1.09<br>(0.96, 1.24)	-	1.05<br>(1.03, 1.07)	1.02<br>(0.99, 1.04)	1.05<br>(0.94, 1.17)
Model 5[e]	1.09<br>(0.80, 1.48)	1.09<br>(0.96, 1.24)	-	1.05<br>(1.03, 1.08)	1.01<br>(0.99, 1.03)	1.05<br>(0.94, 1.17)
Model 6[f]	1.09<br>(0.85, 1.40)	1.07<br>(0.96, 1.20)	-	1.06<br>(1.04, 1.09)	1.01<br>(1.00, 1.02)	1.03<br>(0.96, 1.11)
Model 7[g]	1.11<br>(0.61, 2.03)	1.12<br>(0.96, 1.31)	-	1.03<br>(1.01, 1.05)	1.01<br>(0.99, 1.03)	1.02<br>(0.94, 1.12)

Model 8[h]	1.25 (0.79, 1.97)	1.10 (1.00, 1.21)	-	1.04 (1.03, 1.06)	1.00 (0.99, 1.01)	1.07 (0.97, 1.17)
Model 9[i]	1.17 (0.64, 2.15)	1.14 (0.97, 1.33)	-	1.07 (1.04, 1.10)	1.01 (0.99, 1.04)	1.05 (0.93, 1.17)
Model 10[j]	1.08 (0.80, 1.47)	1.09 (0.96, 1.24)	-	1.05 (1.03, 1.08)	1.01 (0.99, 1.04)	1.05 (0.94, 1.17)
Model 11h[k]	1.06 (0.71, 1.57)	1.20 (0.87, 1.64)	-	1.03 (1.01, 1.04)	1.01 (0.99, 1.02)	1.01 (0.98, 1.03)
Model 12[l]	1.16 (0.80, 1.68)	1.06 (0.93, 1.22)	-	1.06 (1.01, 1.10)	1.02 (1.00, 1.05)	1.11 (0.96, 1.28)
Extreme heat						
Model 1[a]	1.07 (0.73, 1.56)	1.14 (0.97, 1.33)	-	1.24 (1.16, 1.32)	1.07 (1.00, 1.15)	1.05 (0.92, 1.20)
Model 2[b]	1.06 (0.74, 1.52)	1.11 (0.95, 1.29)	-	1.19 (1.12, 1.27)	1.05 (0.99, 1.12)	1.03 (0.94, 1.13)
Model 3[c]	1.27 (0.78, 2.09)	1.14 (0.96, 1.36)	-	1.20 (1.14, 1.27)	1.08 (0.99, 1.18)	1.06 (0.90, 1.24)
Model 4[d]	1.03 (0.81, 1.32)	1.13 (0.97, 1.32)	-	1.24 (1.16, 1.31)	1.08 (1.01, 1.15)	1.06 (0.93, 1.21)
Model 5[e]	1.07 (0.73, 1.58)	1.13 (0.97, 1.32)	-	1.23 (1.16, 1.31)	1.07 (1.00, 1.15)	1.05 (0.92, 1.20)
Model 6[f]	1.26 (0.93, 1.71)	1.10 (0.96, 1.26)	-	1.23 (1.17, 1.30)	1.07 (1.01, 1.13)	1.07 (0.97, 1.19)
Model 7[g]	1.12 (0.57, 2.18)	1.15 (0.95, 1.37)	-	1.16 (1.08, 1.25)	1.07 (0.99, 1.16)	1.06 (0.92, 1.22)
Model 8[h]	1.09 (0.88, 1.35)	1.15 (1.02, 1.29)	-	1.19 (1.13, 1.24)	1.04 (0.98, 1.10)	1.06 (0.95, 1.17)
Model 9[i]	1.12 (0.57, 2.20)	1.20 (1.00, 1.45)	-	1.29 (1.21, 1.38)	1.09 (1.01, 1.18)	1.09 (0.94, 1.26)
Model 10[j]	1.06 (0.73, 1.56)	1.14 (0.97, 1.33)	-	1.24 (1.17, 1.32)	1.07 (1.00, 1.15)	1.05 (0.92, 1.20)
Model 11h[k]	1.20 (0.76, 1.89)	1.28 (0.91, 1.80)	-	1.16 (1.09, 1.24)	1.04 (0.97, 1.11)	1.06 (0.93, 1.20)
Model 12[l]	1.09 (0.70, 1.69)	1.07 (0.92, 1.25)	-	1.23 (1.14, 1.32)	1.16 (1.06, 1.27)	1.08 (0.92, 1.27)

[a] Model 1: Original model: crossbasis with 5 degrees of freedom of exposure and 4 of lag of exposure; maximum lag of 14; time spline with 8 degrees of freedom per year.

[b] Model 2: reducing exposure degrees of freedom to 3.

[c] Model 3: increasing exposure degrees of freedom to 7.

[d] Model 4: reducing the display lag degrees of freedom for 2.

[e] Model 5: increasing the display lag degrees of freedom for 6.

[f] Model 6: reducing the maximum lag to 10.

[g] Model 7: increasing the maximum lag to 18.

[h] Model 8: reducing the degrees of freedom of time to 4 per year.

[i] Model 9: increasing the degrees of freedom of time to 12 per year.

[j] Model 10: including the population log as offset variable.

[k] Model 11: using the minimum temperature instead of the average temperature.

[l] Model 12: using the maximum temperature instead of the average temperature.
